# Investigating the role of the carbon storage regulator A (CsrA) in *Leptospira* spp.

**DOI:** 10.1371/journal.pone.0260981

**Published:** 2021-12-13

**Authors:** Theerapat Phoka, Lenka Fule, Juliana Pipoli Da Fonseca, Thomas Cokelaer, Mathieu Picardeau, Kanitha Patarakul

**Affiliations:** 1 Medical Microbiology, Interdisciplinary and International Program, Graduate School, Chulalongkorn University, Bangkok, Thailand; 2 Institut Pasteur, Biology of Spirochetes Unit, French National Reference Centre for Leptospirosis, Paris, France; 3 Plate-forme Technologique Biomics, Centre de Ressources et Recherches Technologiques (C2RT), Institut Pasteur, Paris, France; 4 Hub de Bioinformatique et Biostatistique – Département Biologie Computationnelle, Institut Pasteur, Paris, France; 5 Department of Microbiology, Faculty of Medicine, Chulalongkorn University, Pathumwan, Bangkok, Thailand; 6 Chula Vaccine Research Center (Chula VRC), Center of Excellence in Vaccine Research and Development, Chulalongkorn University, Bangkok, Thailand; University of Kentucky College of Medicine, UNITED STATES

## Abstract

Carbon Storage Regulator A (CsrA) is a well-characterized post-transcriptional global regulator that plays a critical role in response to environmental changes in many bacteria. CsrA has been reported to regulate several metabolic pathways, motility, biofilm formation, and virulence-associated genes. The role of *csrA* in *Leptospira* spp., which are able to survive in different environmental niches and infect a wide variety of reservoir hosts, has not been characterized. To investigate the role of *csrA* as a gene regulator in *Leptospira*, we generated a *L*. *biflexa csrA* deletion mutant (Δ*csrA*) and *csrA* overexpressing *Leptospira* strains. The Δ*csrA L*. *biflexa* displayed poor growth under starvation conditions. RNA sequencing revealed that in rich medium only a few genes, including the gene encoding the flagellar filament protein FlaB3, were differentially expressed in the Δ*csrA* mutant. In contrast, 575 transcripts were differentially expressed when *csrA* was overexpressed in *L*. *biflexa*. Electrophoretic mobility shift assay (EMSA) confirmed the RNA-seq data in the Δ*csrA* mutant, showing direct binding of recombinant CsrA to *flaB3* mRNA. In the pathogen *L*. *interrogans*, we were not able to generate a *csrA* mutant. We therefore decided to overexpress *csrA* in *L*. *interrogans*. In contrast to the overexpressing strain of *L*. *biflexa*, the overexpressing *L*. *interrogans* strain had poor motility on soft agar. The overexpressing strain of *L*. *interrogans* also showed significant upregulation of the flagellin *flaB1*, *flaB2*, and *flaB4*. The interaction of *L*. *interrogans* rCsrA and *flaB4* was confirmed by EMSA. Our results demonstrated that CsrA may function as a global regulator in *Leptospira* spp. under certain conditions that cause *csrA* overexpression. Interestingly, the mechanisms of action and gene targets of CsrA may be different between non-pathogenic and pathogenic *Leptospira* strains.

## Introduction

*Leptospira* spp. are gram-negative, spiral-shaped bacteria categorized into non-pathogenic and pathogenic strains. Non-pathogenic *Leptospira* spend their entire life in the natural environment. Pathogenic *Leptospira* can survive in the environment and cause leptospirosis in susceptible hosts. It is estimated that 1 million people suffer from severe leptospirosis each year and there are approximately 60,000 deaths, mostly in developing tropical countries [1].

The ability to survive in a wide range of environments is crucial for both pathogenic and non-pathogenic *Leptospira* spp. The pathogenic strains have to complete the zoonotic cycle to live in distinct habitats, including survival in aqueous or terrestrial environment [2], kidneys of their reservoir hosts [3], or target organs of their susceptible hosts [4]. Most transmission occurs when people are exposed to water and soil contaminated by urine of reservoir animals. *Leptospira* can then enter in the host through abraded skin or mucous membrane followed by hematogenous spread to the target organs. The mechanism underlying the long-term survival of pathogenic *Leptospira* under nutrient-poor conditions is not completely understood but biofilm formation may play an important role [5, 6]. Furthermore, omics studies revealed the changes in gene expression profiles in *Leptospira* in response to different environmental conditions such as temperature shift [7], physiologic osmolarity [8], serum exposure [9], iron limitation [10], *in vivo* cultivation on dialysis membrane chamber [11], and in the presence of biofilm [12]. These transcriptome studies highlighted the role of global gene regulation which is a crucial process employed by the bacteria to deal with the changes in the environment. However, due to the lack of efficient genetic manipulation, knowledge of gene regulation is not well understood in *Leptospira* spp. Some regulators have been characterized such as the peroxidase stress regulator PerR [10], the KdpE sensor potassium transport activator [13], DNA repair LexA [14], the sigma factor RpoN [15, 16] and, more recently, the pathogen-specific two-component system LvrAB [17]. Besides these regulators, all leptospiral genomes also possess a gene that encodes the putative CsrA [18–20].

Carbon Storage Regulator A (CsrA) (or its homolog RsmA) is one of the most studied RNA binding proteins in bacteria [21]. This protein is widely conserved in more than 1,500 bacterial species. A transposon mutant of *csrA* in *Escherichia coli* was first reported to display pleiotropic phenotypes including alteration in glycogen accumulation, adhesion ability, and cell size compared to the wild type strain [22]. Due to substantial pleiotropic effects, several omics studies have reported the effect of CsrA on global transcriptomic changes [23–33] (Table 1), showing that CsrA is a global regulator in both gram-positive and gram-negative bacteria. CsrA regulates gene expression at the post-transcriptional level by binding to mRNA targets, and affects mRNA stability and translation [34]. This protein could negatively or positively regulate mRNA expression. For negative regulation, CsrA binds to the Shine-Dalgarno region and prevents ribosome access to the targeted mRNA thus blocks the translation process of the bacteria [35–37]. In addition, CsrA may bind to mRNA targets that overlaps the start codon [38]. For positive regulation, CsrA binds to mRNA target and prevents the target from being cleaved by the RNase [39, 40]. In *E*. *coli*, the consensus sequence of the CsrA binding site is 5’RUACARGGAUGU’3 where the GGA motif is located in a hairpin loop which is a critical binding site for CsrA [41]. The involvement of CsrA in the regulation of various bacterial processes has been reported including carbon metabolism [42], motility [40], biofilm formation [43], quorum sensing [44], stress response [45], as well as virulence-associated traits such as iron acquisition [46], invasion [47], and type III secretion [48].

**Table 1:** Comparison of differentially expressed genes in *csrA* mutants.

Bacterial species	Phylum	Differentially expressed genes (DEGs)		Criteria for DEGs	Reference
		up	down		
*Leptospira biflexa*	Spirochaetes	2	2	log2FC > 0.5 padj <0.05	This study
*Borrelia burgdorferi*	Spirochaetes	86	153	log2FC > 1 and padj <0.05	[23]
*Erwinia amylovora*	Proteobacteria	317	487	log2FC value ≥ 1 and a corrected p-value < 0.05	[24]
*Escherichia coli* K12 MG1655	Proteobacteria	530	390	log2FC > 0.5 and padj <0.05	[25]
*Escherichia coli* (EHEC) O157:H7	Proteobacteria	641	703	FC ≥ 3 and p-values <0.05	[26]
Enteropathogenic *Escherichia coli* (EPEC)	Proteobacteria	97	36	log2FC ≥ 2 and corrected p-value of ≤0.05	[27]
*Salmonella enterica serovar* Typhimurium^1^	Proteobacteria	132	283	log2FC > 0.8 and FDR < 0.05	[28]
*Vibrio cholerae*	Proteobacteria	386	326	FC > 2 and padj value <0.05	[29]
*Helicobacter pylori*	Proteobacteria	3	50	FC > 1.5 and p-values <0.05	[30]
*Legionella pneumophila*	Proteobacteria	236	195	FC > 1.5 and p-values <0.05	[31]
*Serratia* sp. ATCC 39006	Proteobacteria	323	523	FDR threshold of 5%	[33]
*Clostridium acetobutylicum*	Firmicutes	240	312	FDR < 0.001 and |normalized fold_-change| ≥ 2	[32]

^1^Significant change in protein coding RNA in LB medium.

FC, Fold change, FDR, False discovery rate.

Among the phylum of Spirochaetes, CsrA was extensively studied in *Borrelia burgdorferi*, the causative agent of Lyme disease. The *csrA* mutant of *B*. *burgdorferi* showed that there was a decrease in the expression of some virulent-associated proteins and attenuation in the mouse model [49], but these data were not confirmed by another study [50]. CsrA also acts as a repressor of the flagellin protein FlaB [51]. RNA sequencing (RNA-seq) showed that 13% of the genes were differentially expressed in the *csrA* mutant [23].

Our objective was to investigate the role of CsrA in *Leptospira* spp. We generated a *csrA* deletion mutant and *csrA* overexpressing strains to answer this question. In non-pathogenic *L*. *biflexa*, *csrA* was required for growth under starvation conditions. RNA-seq revealed that in rich-nutrient conditions, deletion of *csrA* had minimal impact on global gene regulation. We showed that CsrA is a repressor of flagellin transcripts but no alteration of motility phenotype in both deletion mutant and overexpressing strains was observed. In the pathogen *L*. *interrogans*, overexpression of *csrA* resulted in motility defect and CsrA could bind to flagellin transcripts. Our results demonstrated that the mechanisms of action and gene targets of CsrA appear to be different between pathogenic and non-pathogenic *Leptospira* strains.

## Materials and methods

### Bacterial strains and growth conditions

*Leptospira* spp. were grown in liquid Ellinghausen-McCullough-Johnson-Harris (EMJH) medium (Difco) at 30°C or 1% agar of solid EMJH at 30°C. The saprophyte *Leptospira biflexa* serovar Patoc strain Patoc1 and the pathogen *Leptospira interrogans* serovar Manilae strain L495 were obtained from the French National Reference Center (NRC) for Leptospirosis (Institut Pasteur, Paris, France). *Escherichia coli* strains were grown in Luria-Bertani (LB) medium at 37°C. When needed, an appropriate antibiotic was added to the culture medium. Bacterial strains are listed in Table 2.

**Table 2:** Bacterial strains used in this study.

Strain	Antibiotic Selection	Description
WT *L*. *biflexa* serovar Patoc	No	Control strain
WT *L*. *biflexa* serovar Patoc + pMaORI	Spectinomycin 50 mg/mL	Control strain with empty replicative plasmid
WT *L*. *biflexa* serovar Patoc + pMaORI_P*csrA*_*lb*_	Spectinomycin 50 mg/mL	Overexpressing strain with Prom_*flgN-flgK-flgL-fliW-csrA*_ *csrA*
Δ*csrA L*. *biflexa* serovar Patoc	No (for selection: Kanamycin 100 mg/mL)	*csrA* deletion mutant
Δ*csrA L*. *biflexa* serovar Patoc + pMaORI_P*csrA*_*lb*_	Spectinomycin 50 mg/mL	Complemented strain with Prom_*flgN-flgK-flgL-fliW-csrA*_ *csrA*
Δ*csrA L*. *biflexa* serovar Patoc + pMaORI	Spectinomycin 50 mg/mL	Control strain with empty replicative plasmid
*L*. *interrogans* serovar Manilae WT	No	Control strain
*L*. *interrogans* serovar Manilae WT + pMaORI_P*csrA*_*li*_	Spectinomycin 50 mg/mL	Overexpressing strain with Prom_*flgN-flgK-flgL-fliW-csrA*_ *csrA*
*L*. *interrogans* serovar Manilae WT + pMaORI	Spectinomycin 50 mg/mL	Control strain with empty replicative plasmid
*E*. *coli* DH5α	No	Strain for cloning and plasmid amplification
*E*. *coli* TOP10 thermo	No	Strain for cloning and plasmid amplification
*E*. *coli* Bl-21(DE3) pLysS	No	Strain for recombinant protein production
*E*. *coli* β2163	No	Donor strain for conjugation with *Leptospira* spp.
*E*. *coli* P1	No	Strain for plasmid amplification

For growth curves, the bacteria were grown in EMJH medium until the culture reached exponential phase (OD_420_ ~ 0.1 to 0.2 or 2.5×10^8^ cells/mL). Then, 2×10^6^ bacteria were added into 10 mL of EMJH medium. The cultures were incubated at 30°C, at 30°C with 100 rpm shaking or at 37°C with 200 rpm shaking. One mL of each culture was taken for OD_420_ measurement every 24 h. In order to perform a growth curve in diluted EMJH, *Leptospira* cells were prepared as described above before inoculation into 1/5 EMJH medium diluted in sterile water.

### Allelic exchange mutagenesis of leptospiral *csrA*

A *L*. *biflexa csrA* deletion mutant was generated by allelic exchange. Briefly, a plasmid containing a kanamycin resistance cassette was used to replace the coding sequence of *csrA*, LEPBIa3210, and 0.8 kb sequences that flanked the target gene was synthesized by GeneArt (Life Technologies, Grand Island, NY, USA), pretreated by UV, and used to transform *L*. *biflexa* as previously described [52]. A similar strategy was performed for the *csrA* homolog, LIMLP_17575, in *L*. *interrogans* serovar Manilae. The map of each suicide plasmid is shown in S1 Fig.

To check for a double crossing-over event among the kanamycin-resistant colonies of *L*. *biflexa*, genomic DNA was isolated from exponential phase cultures using a Maxwell 16 cell DNA purification kit and a Maxwell instrument (Promega, Madison, WI), and PCR was performed on DNA extracts with the following primer pairs: 1) Flk_L and Flk_R, and 2) ORF_L and ORF_R.

### Construction of the plasmids and *E*. *coli* β2163 conjugation with *Leptospira* spp.

To construct the plasmids for complementation and overexpression, the *L*. *biflexa* and *L*. *interrogans csrA* genes were cloned into 2 different vectors. We first cloned *csrA* in pMaGro [53] in front of a strong promoter *groES*. We also synthesized a transcription fusion of *csrA* with a promoter of operon *flgN-flgK-flgL-fliW-csrA* by GeneArt (Life Technologies, Grand Island, NY, USA). This fusion was cloned into the SacI and XbaI sites of pMaORI [54]. All pMaORI constructs are shown in S2 Fig.

Conjugation was performed as previously described [55]. Briefly, *E*. *coli* β2163 containing plasmid of interest was incubated with log-phase *Leptospira* on a membrane filter and placed on EMJH plate supplemented with 0.3 mM diaminopimelic acid and incubated for 16–20 h at 30°C. The bacteria were then resuspended in EMJH and spread onto EMJH solid agar plates supplemented with 50 μg/mL spectinomycin. The plates were incubated at 30°C until leptospiral colonies were observed, approximately 1 week for *L*. *biflexa* and 2 weeks for *L*. *interrogans*.

### RNA purification and RT-qPCR

RNA isolation was performed as previously described [56]. Briefly, *Leptospira* spp. were grown until the growth reached exponential phase, OD_420_ ~ 0.1 to 0.2 or ~ 2.5 × 10^8^ cells/mL. The cells were harvested and RNA was extracted using TRIZOL reagent (Thermo Fisher Scientific, Vantaa, Finland) as previously described [56]. RNA pellets were resuspended in UltraPure Dnase/Rnase Free Distilled Water (Thermo Fisher Scientific). Genomic DNA was removed by DNase treatment using the RNase-free Turbo DNA-free turbo kit (Thermo Fisher Scientific) following the manufacturer’s instructions. The 500 ng of RNA were used for cDNA synthesis using iScript^™^ Advanced cDNA Synthesis Kit for RT-qPCR (Bio-Rad Laboratories, Hercules, CA). Quantitative reverse transcription-PCR (RT-qPCR) was performed using SYBR^®^ Green Master Mix (Bio-Rad). The results were expressed as the normalized difference of the threshold cycle (ΔΔCT), using *cysK* and *lipL32* as a reference gene for *L*. *biflexa* and *L*. *interrogans*, respectively. All primers are listed in S1 Table.

### RNA-sequencing

As previously described [57], RNA integrity was examined using the RNA 6000 Nano kit with the Agilent 2100 bioanalyzer (Agilent Technologies, Wilmington, DE) and all samples used for constructing the library had RNA Integrity Number (RIN) scores >8.

The QIAseq FastSelect -5S/16S/23S kit (QIAGEN) was used to deplete ribosomal RNA according to the manufacturer’s instructions. The libraries were built using the TruSeq Stranded mRNA library Preparation Kit (Illumina, USA) following the QIAseq Fastselect -5S/16S/23S protocol recommendations. Quality control of the libraries was made on the Fragment Analyzer. The sequencing of the libraries was performed on the Illumina NextSeq 500 platform using single-end 150bp format. The RNA-seq analysis was performed with Sequana (version 0.9.6) [58]. In particular, we used the RNA-seq pipeline (version 0.9.20, https://github.com/sequana/sequana_rnaseq). The differential expression analysis testing included normalization conducted with DESeq2 [59, 60]. For each comparison, a p-value adjustment (padj) was performed to take into account multiple testing indicating the significance (Benjamini-Hochberg adjusted p-values [61], FDR < 0.05) and the effect size (fold-change) for each comparison. Genes with an adjusted p-value (padj) lower than 0.05 and a log2FC higher or lower than 0.5 were considered differentially expressed. These datasets were deposited into the ArrayExpress database at EMBL-EBI (www.ebi.ac.uk/arrayexpress) under the accession number E-MTAB-10396.

### Measurement of motility, cell length, and velocity

The motility was checked on 0.6% semisolid EMJH medium. Exponential-phase *Leptospira* were diluted in EMJH to obtain OD_420_ = 0.1 as a starter culture. A small divot was gouged into the agar surface into which 2μL or 5μL of the inoculum was pipetted. The plates were incubated for 1 week for *L*. *biflexa* and 2 weeks for *L*. *interrogans*. The diameter of the zone for each colony was measured to the nearest millimeter.

For cell length and velocity measurement, late exponential-phase cultures (OD_420_ ~ 0.5) were diluted in EMJH broth to obtain an appropriate number of cells per field for visualization under a dark-field microscope. For cell length, approximately 100 cells per strain were measured in randomly selected fields by using cellSens software (Olympus, Hamburg, Germany). Velocity measurement was performed by video microscopy as described previously [6]. Approximately 70 cells per strain were recorded over 60 s. Trajectory analysis and speed displacement were calculated using Olympus CellSens software. Statistical analysis of motility, cell length, and velocity was performed using an Unpaired T-Test (Prism 5.03, GraphPad Software). A p-value < 0.05 was defined as statistically significant.

### Recombinant protein production

PCR products of full sequences of *csrA* amplified from *L*. *biflexa* serovar Patoc or *L*. *interrogans* serovar Manilae genomic DNA were cloned into pRSET-C (Invitrogen). The recombinant plasmids were transformed into *E*. *coli* DH5α and verified by DNA sequencing (Macrogen., South Korea). Recombinant proteins with N-terminus 6× His tag was induced in *E*. *coli* BL21 (DE3) pLysS by 1 mM IPTG at 37°C for 4 h. The pelleted bacteria were resuspended in phosphate buffered saline (PBS) pH 7.4 and disrupted using a high-pressure homogenizer (Constant System Ltd., Northants, UK). The soluble fraction was isolated by centrifugation at 15000 ×g at 4°C for 30 min. Protein samples were purified using Ni Sepharose columns (GE Healthcare, Buckinghamshire, UK) and dialyzed with PBS pH 7.4. To check for the purity of the purified recombinant proteins, the proteins were subjected to 15% Sodium Dodecyl Sulfate polyacrylamide gel electrophoresis (SDS-PAGE) and transferred to nitrocellulose membranes. The membranes were blocked with blocking buffer (1% BSA in PBS pH 7.4 plus 0.05% Tween 20, PBST) before the anti-6× His tag monoclonal antibody (1:5000; KPL, MD, USA) was added. The membranes were further incubated with the horseradish peroxidase (HRP)-conjugated goat anti-mouse IgG (secondary antibody). All incubations were performed at room temperature for 1 h. After incubation, washing step was performed with PBST three times for 5 min each. Amersham ECL (GE Healthcare), an HRP substrate, was added and incubated for 1 min at room temperature before the membrane was exposed to a CCD camera (Bio-Rad) for chemiluminescent signal reading.

### Electrophoretic mobility shift assay (EMSA)

All RNA probes were synthesized (Thermo Fisher Scientific) as follows, LEPBIa_1872 WT 5’UGGACACACAGGAGGGUGUGAC’3, LEPBIA_1872 Mut 5’UGGACACACAAAAGGGUGUGAC’3, and LIMLP_07475 5’AUCGGAUUCAAGGAGGAACCGA’3.

EMSA was performed according to the manual of LightShift^™^ EMSA Chemiluminescent RNA Kit (Thermo Fisher Scientific). Briefly, the binding reaction was prepared. Each binding reaction consisted of 1X binding buffer (10mM HEPES pH 7.3, 20 mM KCL, 1 mM MgCl_2_, and 1 mM DTT) 1 nM of biotinylated-RNA (LEPBIa_1872 WT, LEPBIa_1872 Mut, or LIMLP_07475), 7.5% glycerol, 10 mM DTT, 0.2 μg/μL Yeast tRNA, and various concentrations of rCsrA in a total volume of 20 μL. The binding reaction was incubated at 37°C for 30 min. After incubation, loading buffer was added into each reaction and separated on 10% native PAGE for 1 h at 100V. The reaction was transferred onto a nylon membrane, crosslinked with UV for 1 min, blocked for 15 min with a blocking buffer, and washed once with washing buffer. A 1:300 stabilized Streptavidin-HRP in a blocking buffer was added and incubated for 15 min. The membrane was washed 5 times with washing buffer and incubated for 5 min with a substrate equilibration buffer. The membrane was incubated for 5 min in HRP substrate before chemiluminescent signal reading. For competitive EMSA assay, the binding reaction was prepared as described above except rCsrA concentration was fixed at 800 nM while unlabeled RNA (LIMLP_07475) was added to the solution at the final concentrations ranged from 0.8 nM to 8 μM (10-fold serial dilution).

## Results

### Genetic organization of the *csrA* locus in *L*. *interrogans* and *L*. *biflexa*

**Fig 1:**
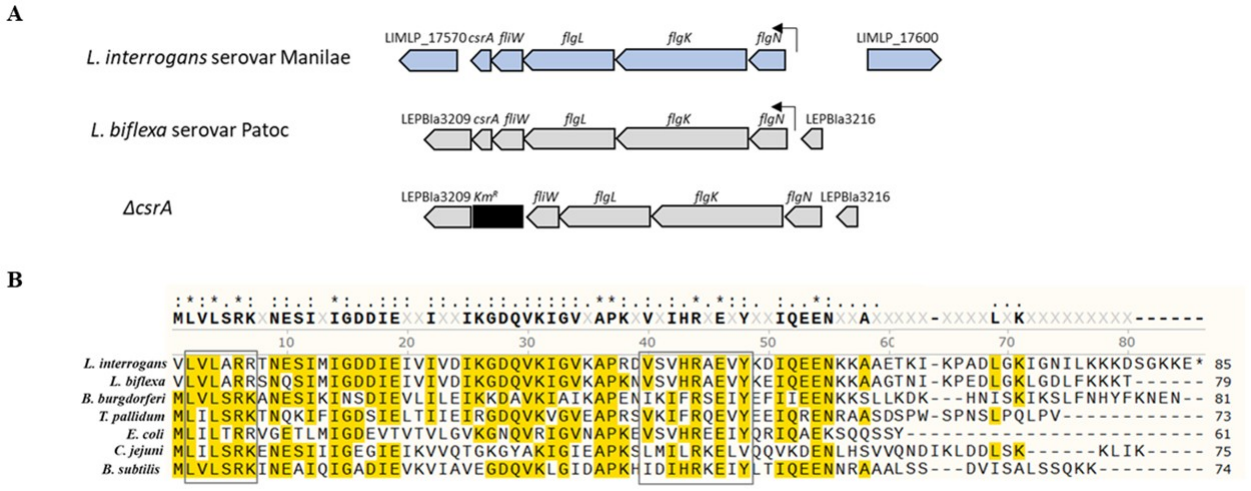
*csrA* operon in *Leptospira* spp. (A) A genetic organization of *csrA* in *Leptospira* spp. The arrangement of the genes in the *csrA* operon in *L*. *interrogans*, *L*. *biflexa*, and *L*. *biflexa* Δ*csrA* are shown. (B) The alignment of the amino acid sequences of CsrA in *L*. *biflexa* serovar Patoc and *L*. *interrogans* serovar Manilae strains used in this study was performed in comparison with CsrA from other bacteria. (*) represents conserved amino acid and the square boxes indicate conserved residues that are important for RNA binding in *E*. *coli* [62]. Sequences highlighted in yellow indicate the conserved residues.

The *csrA* locus is conserved in the pathogen *L*. *interrogans* serovar Manilae and the saprophyte *L*. *biflexa* serovar Patoc; the *csrA* forms with the flagellar genes to develop an operon-like structure (Fig 1A). This operon consists of 5 consecutive genes: *flgN*, *flgK*, *flgL*, *fliW* and *csrA*. The genes *flgK* and *flgL* encode putative flagellar hook-associated proteins, and *flgN* encodes a putative chaperone for FlgK and FlgL. The gene *fliW* encodes a putative post-transcriptional regulator of flagellin. There is a 200-bp intergenic region located upstream of *flgN*, the first gene of the operon, suggesting that there is a putative promoter region. The CsrA of *L*. *biflexa* and *L*. *interrogans* share >88% sequence identity, while both share ~50–60% similarity compared with CsrA from other bacteria. The amino acid alignment of leptospiral CsrA shows conserved sequences (highlighted in yellow) and 2 domains (in square boxes) reported as critical for RNA binding in *E*. *coli* [62] (Fig 1B). In addition, leptospiral CsrA is slightly longer than that of other bacteria due to additional C-terminal amino acid residues.

### Allelic exchange mutagenesis and complementation of *csrA* in *L*. *biflexa*

**Fig 2:**
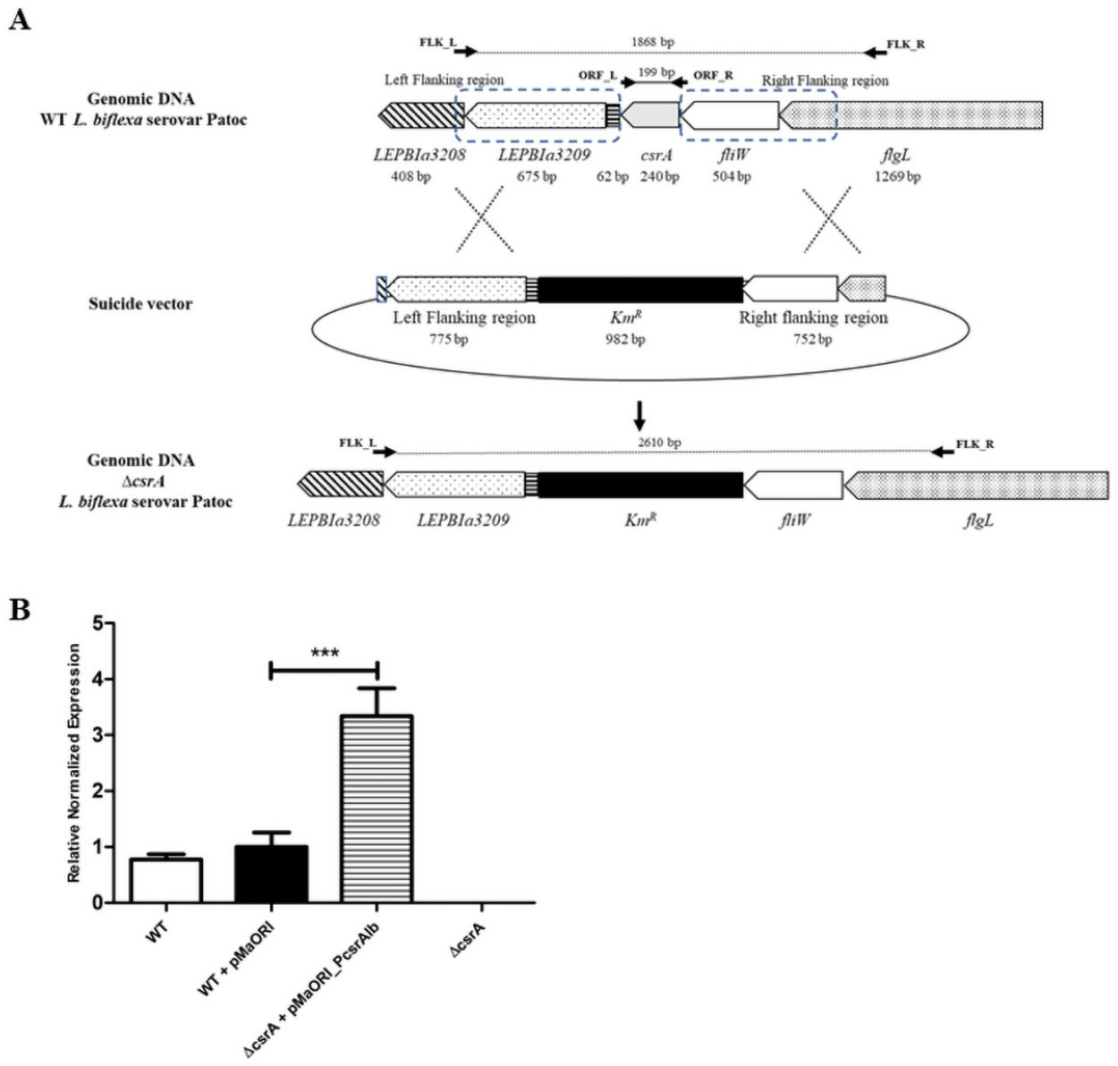
Allelic exchange of *csrA* in *L*. *biflexa*. (A) Schematic representation of homologous recombination. To generate a *csrA* mutant, *L*. *biflexa* serovar Patoc was electroporated with a suicide vector containing the *csrA* locus where *csrA* was replaced by a kanamycin resistance cassette (*Km*^*R*^). Genes and non-coding regions with their sizes (bp) are indicated. The flanking regions of *csrA* used for homologous recombination are indicated by the dashed line square. Arrows indicate primers used for the confirmation of double crossing-over events. (B) *csrA* expression in *L*. *biflexa* strains was determined by RT-qPCR. Results obtained from 3 independent cultures were presented as relative fold changes ±SEM using *cysK* gene for normalization. (***) indicates p-value <0.001.

In this study, the suicide plasmids containing the *L*. *biflexa csrA* and *L*. *interrogans csrA* were disrupted by a kanamycin-resistance cassette (*Km*^*R*^) and transformed in saprophyte *L*. *biflexa* and the pathogen *L*. *interrogans*, respectively. Transformant colonies were only obtained in *L*. *biflexa*. We were unable to get transformant colonies from *L*. *interrogans* after 5 attempts. Among the 16 randomly selected kanamycin-resistant colonies of *L*. *biflexa*, 5 (31%) produced a ~2.6 kb PCR product with Flk primers which indicated that *csrA* was successfully replaced with kanamycin-cassette by a double crossing-over event; for the other colonies, the kanamycin-cassette was successfully replaced by a single cross-over event (Fig 2A and S3A Fig). To further confirm the deletion of *csrA* in the double-crossover mutants, primers ORF-R and ORF-L were also used. While the WT produced the expected size of 199-bp PCR product, approximately 1-kb PCR products were obtained from the transformants with allelic exchange (S3B Fig). These results indicated that there was a successful allelic exchange of *csrA* in *L*. *biflexa* which was designated as Δ*csrA*.

In order to complement the Δ*csrA*, our first attempt was to express the wild-type *csrA* under a strong promoter of *Leptospira*, P_*groES*_, but no transconjugant was obtained. We hypothesized that the excess level of CsrA may be toxic to *Leptospira*. Therefore, we expressed *csrA* under the control of its native promoter, which is the promoter of the operon containing *flgN*, *flgK*, *flgL*, *fliW*, and *csrA (*Fig 1A). The resulting plasmid was used for complementation in Δ*csrA*. RT-qPCR revealed that the relative fold change of *csrA* in the complemented strain (Δ*csrA*+pMaORI_P*csrA*_*lb*_) was 3.33-fold higher compared with WT+pMaORI (Fig 2B), indicating overexpression of *csrA*. In addition, RT-qPCR was unable to detect the expression of *csrA* in the Δ*csrA*+pMaORI, confirming the successful deletion of *csrA* in *L*. *biflexa*.

### Phenotype analysis of the Δ*csrA L*. *biflexa*

#### Effects of *csrA* on growth and motility

**Fig 3:**
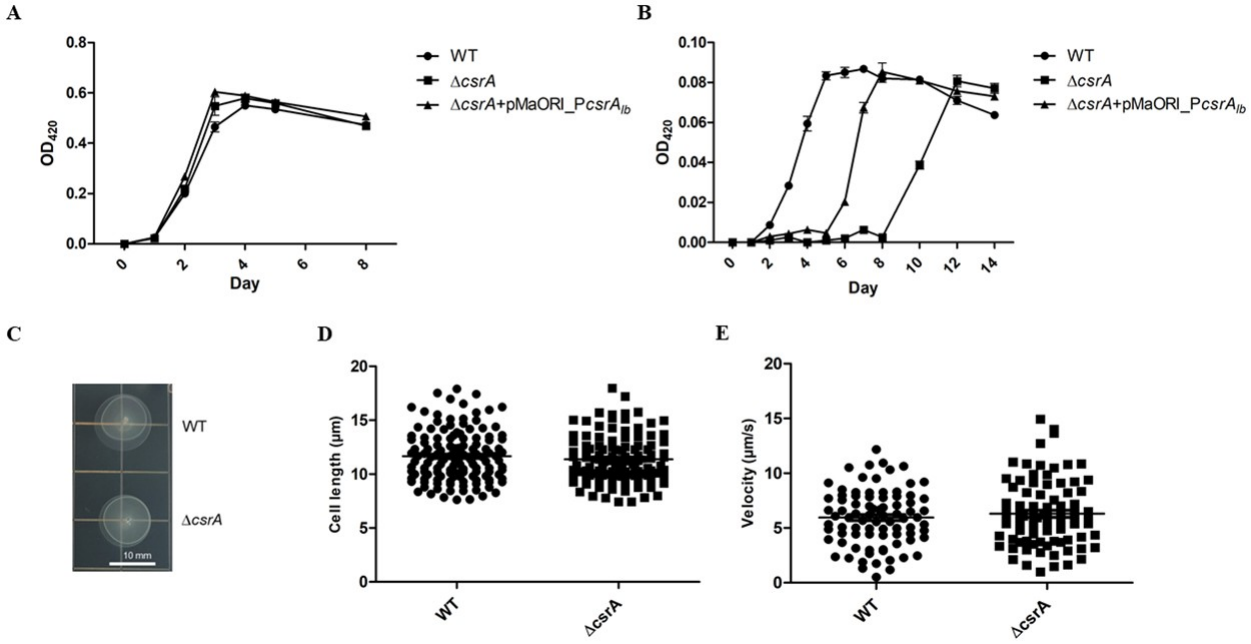
Phenotype analysis of the Δ*csrA L*. *biflexa*. To investigate the effect of *csrA* on growth, 2×10^6^ cells of each bacterial strain were grown in 10 mL of (A) regular EMJH and (B) 5-fold diluted EMJH. OD_420_ measurement for growth was performed every 24 h. Results obtained from 3 independent experiments are expressed as Mean ± SEM. (C) Soft agar assay of WT and Δ*csrA*. *Leptospira* were inoculated onto 0.6% semisolid EMJH plate and incubated at 30°C for one week before measuring the diameter of each colony. The late exponential phase of *Leptospira* grown in EMJH medium were measured for (E) cell length and (D) velocity under a dark-field microscope using cellSens software (OLYMPUS).

The growth curve of WT, Δ*csrA*, and Δ*csrA+*pMaORI_P*csrA*_*lb*_ in regular EMJH were comparable (Fig 3A), suggesting that *csrA* was not essential for growth in *L*. *biflexa*. However, we found that Δ*csrA* displayed poor growth in 5-fold diluted EMJH compared to the WT (Fig 3B). Complementation of the Δ*csrA* partially restored the wild-type phenotype under starvation conditions (Fig 3B).

We performed soft agar assays to determine the motility of Δ*csrA*, but we did not find any differences between WT and Δ*csrA* (Fig 3C). Consistent with soft agar results, we did not find any difference in cell length or velocity in liquid EMJH between WT and Δ*csrA* (Fig 3D and 3E). In addition, the motility behavior of Δ*csrA* observed under the dark-field microscope was similar to WT (data not shown).

#### RNA-sequencing

**Fig 4:**
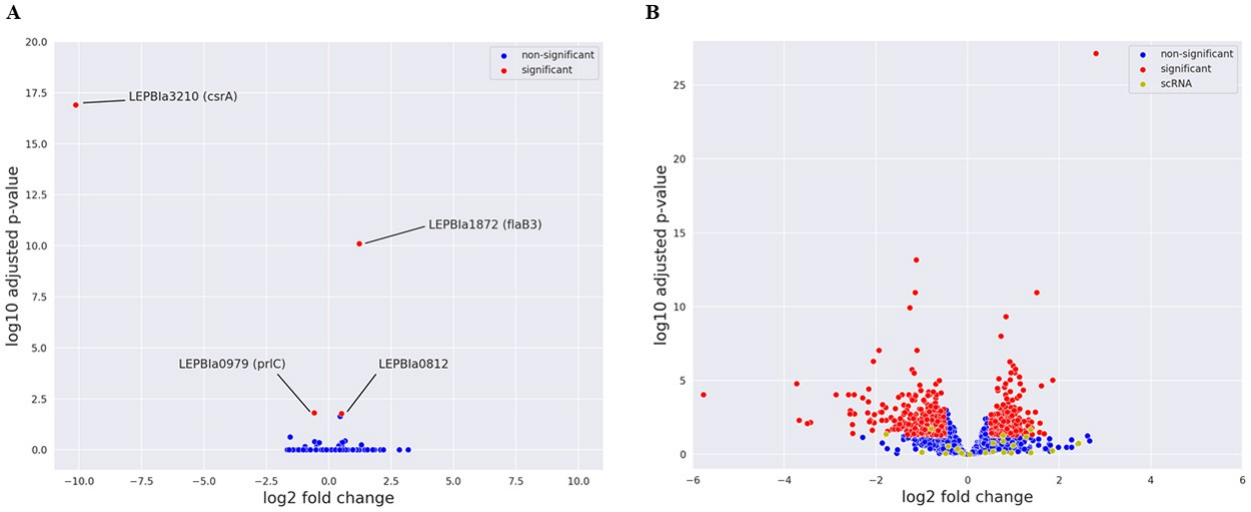
RNA-sequencing. The up- and downregulated genes in Δ*csrA* or Δ*csrA*+pMaORI_P*csrA*_*lb*_ compared with WT are shown in the Volcano analysis. (A) Comparison between Δ*csrA* and WT and (B) Comparison between Δ*csrA*+pMaORI_P*csrA*_*lb*_ and WT. Red dots indicated up- or downregulated genes with log2FC > ± 0.5 and adjusted p-value (padj) < 0.05. Representative genes are labeled. Blue and yellow dots indicate non-differentially expressed genes and scRNA, respectively.

To investigate the role of *csrA* as a global gene regulator in *L*. *biflexa*, RNA-seq was performed on exponential-phase cultures of WT, Δ*csrA*, and Δ*csrA*+pMaORI_P*csrA*_*lb*_. With log2FC > ±0.5 and padj<0.05 as the criteria for differentially expressed gene (DEG), only 3 genes, not including *csrA*, were differentially expressed in Δ*csrA* compared with the WT strain which is accounting for less than 0.1% of total ORF (3 in 3730) (Fig 4A, Table 3 and S2 Table). Two genes were significantly upregulated in Δ*csrA; LEPBIa_1872* (encodes a flagellin protein FlaB3), and *LEPBIa_0812* (encodes putative acyltransferase) by 2.331-, and 1.423-fold, respectively, while *LEPBIa0979* (encodes oligopeptidase A) was 0.668-fold downregulated (Table 3). Furthermore, the level of *LEPBIa_1872* was restored to WT level of Δ*csrA*+pMaORI_P*csrA*_*lb*_, suggesting that *LEPBIa_1872* should be a specific gene target of *L*. *biflexa* CsrA. In contrast, the complementation of Δ*csrA* could not restore wild-type expression of *LEPBIa_0812* and *LEPBIa0979*, suggesting that these genes are not putative gene targets of CsrA (Table 3).

**Table 3:** Selected differentially expressed genes in Δ*csrA* and Δ*csrA*+pMaORI_P*csrA*_*lb*_ compared to WT.

ORF ID^a^	Gene^a^	Product^a^	COGs^a^	Δ*csrA* vs WT FC	Δ*csrA* vs WT padj	Δ*csrA*+pMaORI_P*csrA*_*lb*_ vs WT FC	Δ*csrA*+pMaORI_P*csrA*_*lb*_ vs WT padj	Δ*csrA* vs Δ*csrA*+pMaORI_P*csrA*_*lb*_ FC	Δ*csrA* vs Δ*csrA*+pMaORI_P*csrA*_*lb*_ padj	FC (RT-qPCR)^b^	
										Δ*csrA* vs WT	Δ*csrA*+pMaORI_P*csrA*_*lb*_ vs WT
LEPBIa1872	*flaB3*	Flagellar filament 35 kDa core protein	N	2.331	7.95E-11	0.919	0.624	2.537	8.83E-14	4.26*	1.68
LEPBIa0812		Putative acyltransferase, MBOAT family; putative membrane protein	M	1.423	0.016	1.785	4.81E-10	0.797	0.091	2.16*	1.97*
LEPBIa0979	*prlC*	Oligopeptidase A	E	0.668	0.015	0.458	6.82E-14	1.455	0.005	0.52	0.66
LEPBIa3210	*csrA*	Carbon storage regulator homolog	T	0.001	1.23E-17	1.848	0.043	0	3.16E-21	U	3.33*
LEPBIa2344	*groL*	GroEL protein, Hsp60 family	O	0.858	0.994	0.136	8.97E-05	6.288	0.001	ND	ND
LEPBIa2449	*clpB*	Chaperone ClpB	O	0.716	0.994	0.169	0.001	4.236	0.034	ND	ND

^**a**^ ORF ID, Gene, Product, and COG are according to *Leptospira biflexa* serovar Patoc. Patoc 1 was obtained from MicroScope Microbial Genome Annotation & Analysis Platform; https://mage.genoscope.cns.fr/microscope/home/index.php.

^**b**^ Relative fold change (FC) of each gene obtained by RT-qPCR experiments.

(*) indicates significant expression level with p <0.05.

U, undetectable. ND, not determined.

While a few differentially expressed genes were found in Δ*csrA*, 575 transcripts consisting of 569 genes (15% of total ORF), 4 ncRNA, and 2 23S rRNA were differentially expressed in the Δ*csrA* complemented strain compared with WT transcriptome (Fig 4B, S4 Fig and S2 Table). The gene *csrA* (*LEPBIa_3210*) was significantly up-regulated (1.84-fold), further confirming the upregulation of *csrA* observed by RT-qPCR (Fig 2B and Table 3). Among the 569 genes, *clpB* (*LEPBIa_2449*) and *groL* (*LEPBIa2344*), known genes involved in general stress response, are one of the most strongly downregulated genes (Table 3 and S2 Table), indicating that overexpression of *csrA* may induce stress conditions in *L*. *biflexa*. Complete set of ORF expression is shown in S2 Table.

RT-qPCR was performed to validate the RNA-seq results. As shown in Table 3, the significant upregulation of *LEPBIa_0812* and *LEPBIa_1872* was confirmed in Δ*csrA*, while *LEPBIa_0979* was not differentially expressed by RT-qPCR. The restoration of *LEPBIa_1872* in complemented strain was confirmed by RT-qPCR, further confirming this gene as a specific target of CsrA.

#### *FlaB* gene as a potential target of CsrA in *L*. *Biflexa*

**Fig 5:**
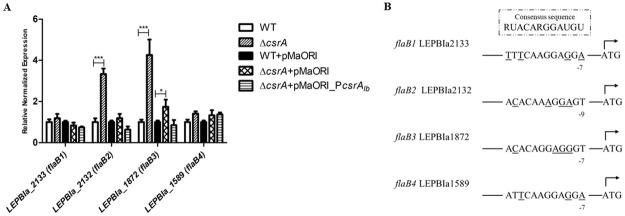
*FlaB* genes expression of *L*. *biflexa*. (A) Expression of *L*. *biflexa flaB* genes by RT-qPCR, RNAs were prepared from 3 independent cultures of each leptospiral strains. Results are presented as relative fold changes ±SEM using *cysK* for normalization. (*), (**), and (***) indicate p-value <0.05, <0.01 and <0.001, respectively. For statistical analysis, Δ*csrA* was compared to WT; Δ*csrA*+pMaORI or Δ*csrA*+pMaORI_P*csrA*_*lb*_ was compared to WT+pMaORI. (B) Analysis of *flaB* 5’ untranslated regions of *L*. *biflexa* serovar Patoc. The gene and distances to the start codon are indicated. Underlined letters represent mismatched nucleotides compared with the consensus sequence.

Because *L*. *biflexa* has 4 *flaB* genes, the effect of *csrA* on the relative expression of these *flaB* genes was determined (Fig 5A). RT-qPCR confirmed an upregulation of *flaB3* in Δ*csrA* and the expression level of *flaB3* was restored in the complemented strain. We also found that *flaB2* (*LEPBIa_2132*) was significantly upregulated in Δ*csrA* and its expression was restored in the complemented strain (Fig 5A). These results indicated that *flaB2* and *flaB3* are potential CsrA targets. The upregulation of both genes was correlated with RNA-seq of Δ*csrA*, of which only *flaB3*, not *flaB2* was differentially expressed (S2 Table).

**Fig 6:**
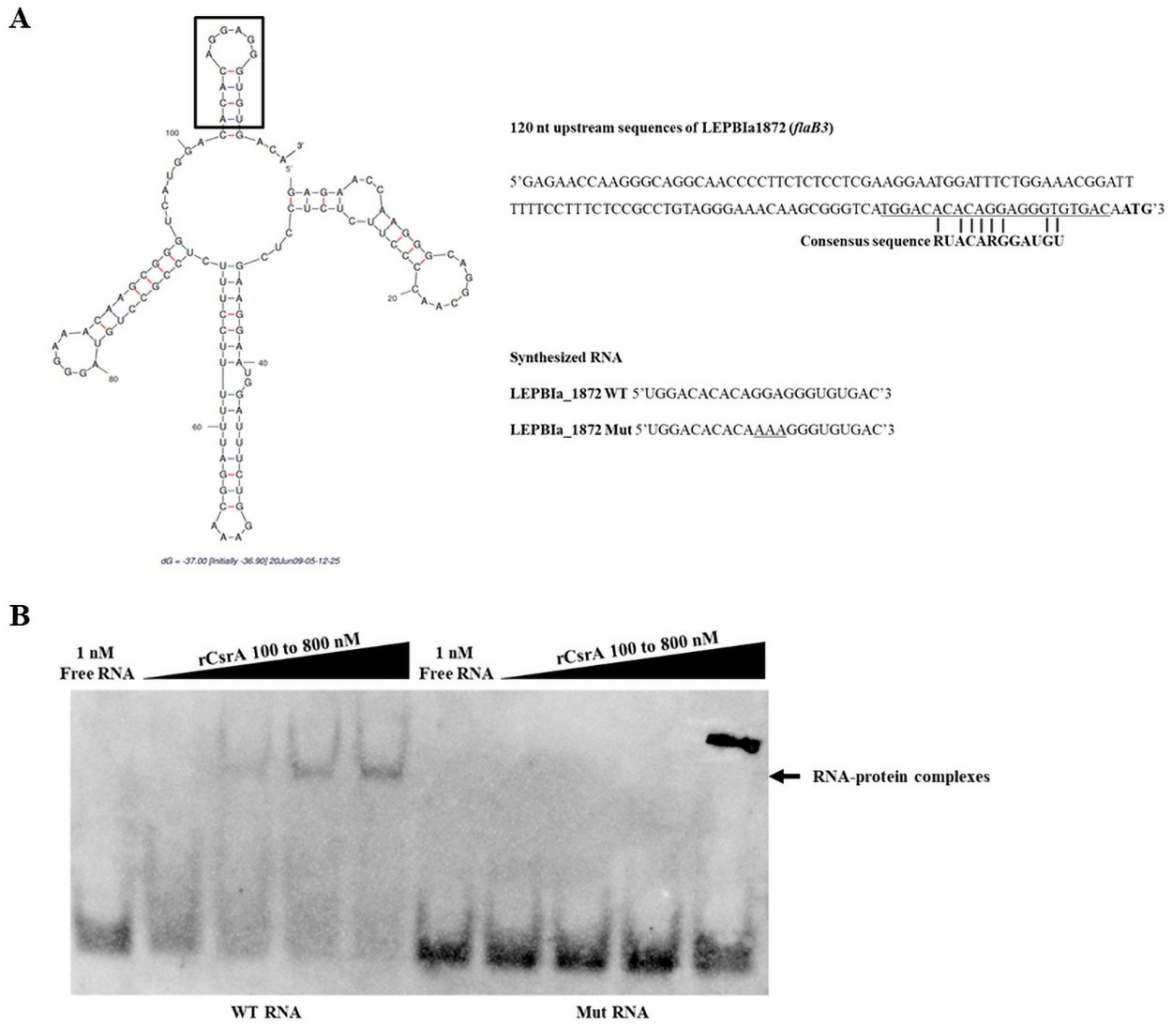
*FlaB* gene as a potential target of CsrA in *L*. *biflexa*. (A) Secondary structure of 120-nucleotide 5’ untranslated region of *LEPBIa1872* (*flaB3*) was predicted using MFOLD [64]. The putative CsrA binding site is shown and the start codon (ATG) is indicated in bold letters. The sequences of the synthesized 5’ biotinylated RNAs of *LEPBIa_1872* WT and *LEPBIa_1872* Mut probes for *flaB3* are shown. (B) Electrophoretic mobility shift assay (EMSA), 1 nM biotinylated RNA of either *LEPBIa_1872* WT or *LEPBIa_1872* Mut probes were incubated with different concentrations of rCsrA of *L*. *biflexa*. The reaction solution was subjected to 10% native PAGE, transferred to a nylon membrane, probed with HRP-conjugated streptavidin, and detected for chemiluminescent signal after the detection reagent was added.

CsrA regulates its targets by binding to their upstream sequences overlapping the Shine Dalgarno sequence [63]. The consensus sequence of the CsrA binding site is 5’RUACARGGAUGU’3 [41]. The upstream sequence analysis of *flaB2* and *flaB3* showed potential CsrA binding sites with 4 nucleotide mismatches compared with the consensus sequence (Fig 5B and S3 Table). The putative binding sites were similar in terms of nucleotide composition and sequence order, 5’ACACAAAGGAGT’3 for *flaB2* and 5’ACACAGGAGGGT’3 for *flaB3*. The Shine Dalgarno sequence (5’AGGAGG’3) was present in the upstream region of *flaB3*, but not *flaB2*, suggesting that *flaB3* might be more promising to be a CsrA target. Secondary structure prediction of 120 nucleotides upstream of *flaB3* revealed that the possible binding site formed a GGA motif-containing hexaloop and localized 7 nucleotides before the start codon (Fig 6A). These findings strongly suggest that *flaB3* is a specific CsrA target in *L*. *biflexa*. Therefore, only the putative CsrA binding site of *flaB3* was selected for further verification.

To confirm the interaction of *L*. *biflexa* CsrA and *flaB3* transcripts *in vitro*, N-terminal 6× His-tag recombinant CsrA protein (rCsrA) of *L*. *biflexa*, with an approximate molecular weight of 14 kDa, was produced in *E*. *coli* (S5 Fig). Electrophoretic mobility shift assay (EMSA) was performed to investigate the interaction between *L*. *biflexa* rCsrA and synthesized 22-nucleotide RNA probe upstream of *flaB3*. Because GGA is a known critical binding site of CsrA, the interaction between rCrsA and GGA motif-containing LEPBIa_1872 WT probe was compared to AAA-containing LEPBIa_1872 Mut probe (Fig 6A). The rCsrA could bind to the WT probe in a dose dependent manner whereas no interaction was observed between rCsrA and the Mut probe (Fig 6B), indicating that GGA motif was critical for *L*. *biflexa* CsrA binding. Therefore, CsrA regulates *flaB3* by binding to its upstream sequence at the GGA motif.

### Overexpression of *csrA* in *Leptospira* spp.

**Fig 7:**
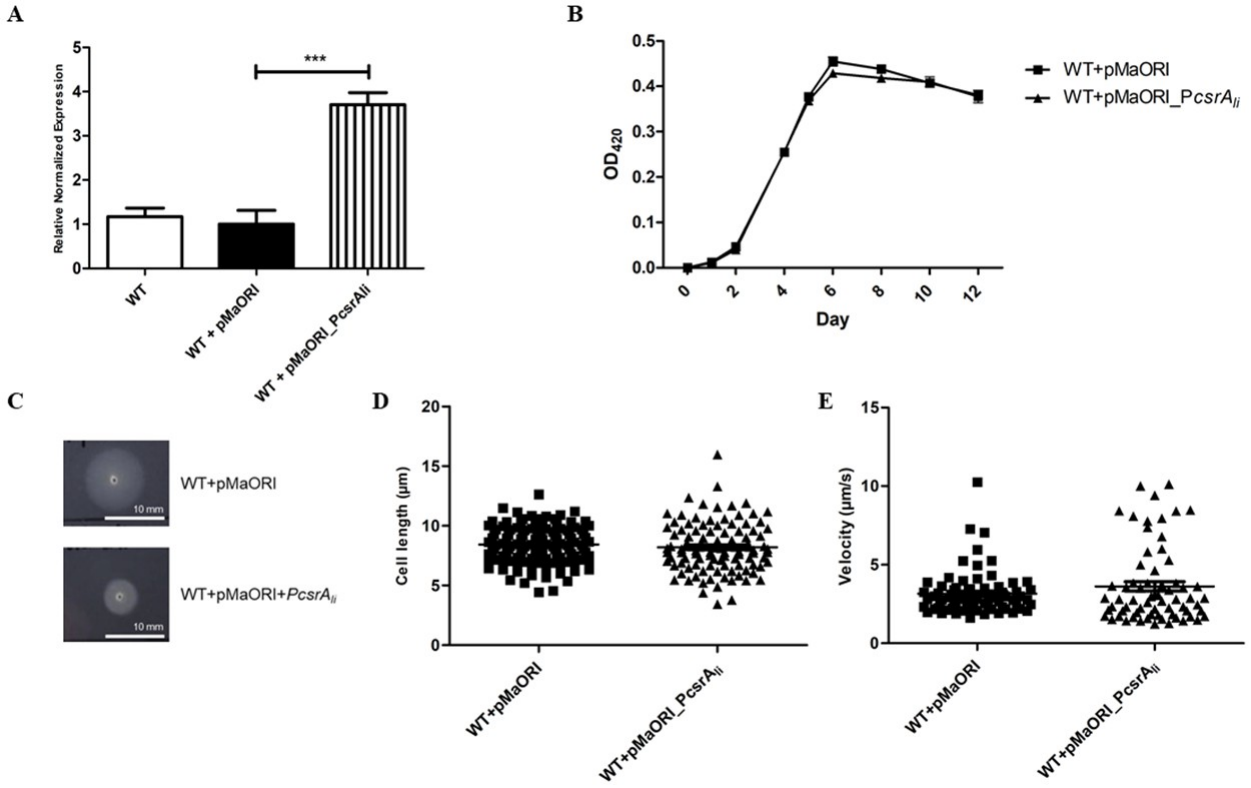
Overexpression of *csrA* in *L*. *interrogans*. (A) Overexpression of *csrA* in *L*. *interrogans*. To confirm overexpression of *csrA*, RNA was extracted from each *Leptospira* strain and then subjected to RT-qPCR. Results obtained from 3 independent cultures were presented as relative fold changes ± SEM. *LipL32* was used for normalization. (***) indicates p-value < 0.001. (B) Growth curve of *L*. *interrogans*. The 2×10^6^ cells of each bacterial strain were grown in 10 mL of regular EMJH. OD_420_ measurement for growth was performed every 24 h. Results obtained from 3 independent experiments are expressed as Mean ± SEM. (C) Soft agar assay of *L*. *interrogans*. *Leptospira* at OD_420_ = 0.1 were inoculated onto 0.6% semisolid EMJH plates and incubated at 30°C. (D) Measurement of cell length of *L*. *interrogans* (E) Measurement of velocity of *L*. *interrogans*. Late exponential phase of *Leptospira* grown in EMJH medium were measured for cell length and velocity under a dark-field microscope using cellSens software (OLYMPUS).

To generate *csrA* overexpressing strains of *L*. *biflexa* and *L*. *interrogans*, we first overexpressed *csrA* of each strain under the control of the promoter of *L*. *interrogans groES*, which previously showed to be a strong promoter [53]. Consistent with the results observed in the complementation experiment, no colony was obtained for both *L*. *interrogans* and *L*. *biflexa*. In contrast, conjugation with the empty replicative plasmid resulted in hundreds of colonies for both *L*. *interrogans* and *L*. *biflexa*. We therefore overexpressed *csrA* under its native promoter, the promoter of the operon *flgN-flgK-flgL-fliW-csrA*. Numerous spectinomycin resistant colonies were then obtained from both leptospiral strains. RT-qPCR showed that the relative fold change of *csrA* was 3.70-fold higher in *L*. *interrogans* (Fig 7A) and 18.77-fold higher in *L*. *biflexa* (S6A Fig) compared to its parental WT strain.

The *csrA* overexpressing strain of both *L*. *interrogans* (WT+pMaORI_P*csrA*_*li*_) and *L*. *biflexa* (WT+pMaORI_P*csrA*_*lb*_) had a growth rate similar to WT in regular and 5-fold diluted EMJH (Fig 7B and S6B and S6C Fig). These results suggested that overexpression of *csrA* did not affect the growth of *Leptospira*.

To investigate the effect of *csrA* overexpression on motility, the soft agar assay and measurement of cell length and motility were performed. We found that the motility of the WT+pMaORI_P*csrA*_*lb*_ was not deficient (S6D–S6F Fig), which was consistent with the results observed in Δ*csrA*. In contrast, overexpression of *csrA* in *L*. *interrogans* had poor motility on soft agar (Fig 7C); however, the cell length and velocity were not significantly different from WT (Fig 7D and 7E).

### Overexpression of *csrA* had a distinct effect on *flaB* expression

**Fig 8:**
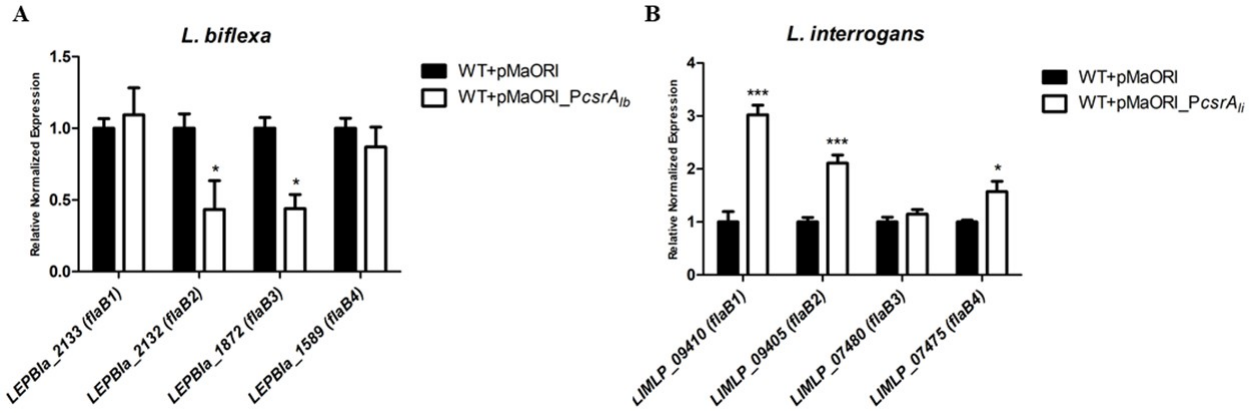
Effect of overexpressed CsrA on *flaB* expression. Expression of 4 *flaB* genes in *Leptospira* strains by RT-qPCR. RNAs were prepared from 3 independent cultures of each leptospiral strain and used for RT-qPCR. Results are presented as relative fold changes ± SEM using *cysK* and *lipL32* for normalization in *L*. *biflexa* and *L*. *interrogans*, respectively. (*), (**) and (***) indicate p-value < 0.05, < 0.01 and < 0.001, respectively.

The relative expression levels of the 4 *flaB* genes in *csrA* overexpressing strains of both *L*. *biflexa* and *L*. *interrogans* were investigated. In WT+pMaORI_P*csrA*_*lb*_, *flaB2* and *flaB3* were significantly downregulated by 0.433- and 0.439-fold change (Fig 8A), respectively, which is in agreement with the data in Δ*csrA* showing that CsrA acts as a repressor in *L*. *biflexa*. In contrast, the overexpressing strain of *L*. *interrogans* showed poor motility on soft agar plates and had a significant upregulation of *flaB1*, *flaB2*, and *flaB4* by 3.02-, 2.10-, and 1.57-fold change, respectively (Fig 8B), suggesting that CsrA might be involved in transcriptional activation of flagellin genes in *L*. *interrogans*.

**Fig 9:**
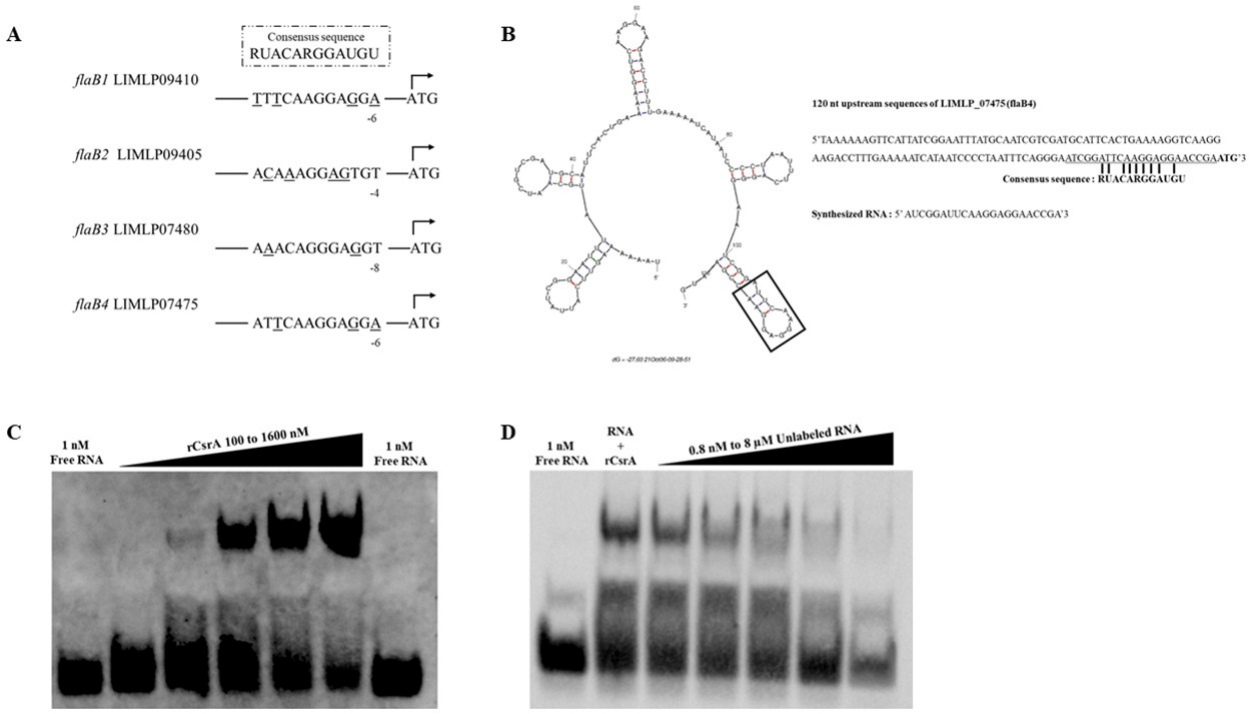
*L*. *interrogans* CsrA regulated *flaB* expression. (**A**) Analysis of *flaB* 5’ untranslated regions of *L*. *interrogans* serovar Manilae. The genes and distances to the start codon are indicated. Underlined letters represent mismatched nucleotides compared to the consensus sequence. (**B**) Secondary structure of 120-nucleotide 5’ untranslated region of LIMLP07475 (FlaB4) was predicted using MFOLD [64]. The putative CsrA binding site is shown and the start codon (ATG) is indicated in bold letters. The sequences of the synthesized 5’ biotinylated RNA of LIMLP_07475 is shown. (**C**) Electrophoretic mobility shift assay (EMSA), 1 nM biotinylated RNA of LIMLP_07475 was incubated with different concentrations of *L*. *interrogans* rCsrA. The reaction solution was subjected to 10% native PAGE, transferred to a nylon membrane, probed with HRP-conjugated streptavidin, and detected for chemiluminescent signal after the detection reagent was added. (**D**) Competitive EMSA, biotinylated RNA of LIMLP_07475 and rCsrA concentration were fixed at 1 nM and 800 nM, respectively. Unlabeled LIMLP_07475 was added in the reaction concentration range from 0.8 nM to 8 μM.

Analysis of 5’ untranslated region of *L*. *interrogans flaBs* revealed putative CsrA binding sites in *flaB1*, *flaB2*, *flaB3*, and *flaB4* (Fig 9A). Among 3 upregulated *flaB* genes, *flaB4* is more likely a CsrA target because of the highest match of its upstream region to the consensus sequence (Fig 9B and S3 Table). Secondary structure prediction of 120 nucleotides upstream of *flaB4* (LIMLP_07475) revealed a GGA motif-containing hexaloop locating 6 nucleotides before the start codon (Fig 9B). Therefore, *flaB4* was selected for further binding studies. Recombinant CsrA protein of *L*. *interrogans* was produced (S5 Fig) and used for EMSA. As expected, rCsrA bound to *flaB4* upstream in a dose-dependent manner (Fig 9C). The specificity was further confirmed by competitive EMSA (Fig 9D). Our results not only demonstrated that CsrA of *L*. *interrogans* is an RNA-binding protein but also showed *flaB4* as a specific target.

## Discussion

*Leptospira* spp. are ubiquitous bacteria found as free-living saprophytes in environmental water and soil or as pathogens excreted in the urine of asymptomatic hosts to cause disseminated infections in both humans and animals. Global gene regulators are required for their rapid adaptation to environmental changes. However, the knowledge of gene regulation in *Leptospira* remains limited. We found that *csrA* homolog, a well-characterized post-transcriptional global regulator, is present in all available leptospiral genomes. The leptospiral *csrA* is located inside an operon of genes involved in the flagellum biosynthesis (Fig 1A) like other bacteria [65]. Moreover, the *csrA* operon of both *L*. *biflexa* and *L*. *interrogans* are in synteny with *csrA* operons of other spirochete bacteria including *B*. *burgdorferi* and *T*. *pallidum* [66]. In gamma-proteobacteria, non-coding RNA (ncRNA), such as *csrB* [67] and *csrC* [68], modulates CsrA function. In epsilon-proteobacteria and firmicutes that have no gene encoding ncRNA antagonist, FliW was reported as the protein antagonist of CsrA [69, 70]. The *Leptospira* genomes do not possess *csrB* and *csrC* homologs but *fliW* is located adjacent to *csrA* (Fig 1A). Thus, FliW may function as the leptospiral CsrA antagonist. A CsrA-like ncRNA, which could regulate leptospiral CsrA activity, was also identified in *L*. *biflexa* [12].

While generation of *csrA* deletion was feasible in *L*. *biflexa*, we were unable to delete *csrA* in *L*. *interrogans*. Because targeted mutation particularly in pathogenic *Leptospira* is difficult and inefficient, a limited number of virulence genes have been confirmed [71]. Likewise, the present study could not successfully generate a deletion mutant in *L*. *interrogans*. Alternatively, *csrA* may have an essential role in the viability of *L*. *interrogans* but not in *L*. *biflexa*. The *csrA* mutant of *Salmonella* Typhimurium showed severe growth defect compared to its parental strain [47]. Moreover, CsrA might be toxic to *Leptospira* because we were unable to obtain any transconjugant in both *L*. *biflexa* and *L*. *interrogans* when *csrA* was fused to a strong promoter.

Metabolism is one of the common phenotypes regulated by CsrA in many bacteria [72–76]. For instance, *E*. *coli* CsrA regulates the carbon starvation gene, *cstA*, which plays a role in peptide transport during carbon starvation [76]. Another study reported a strong activity of CsrA during iron-limited condition [46]. In regular EMJH, growth curves of Δ*csrA* and WT+pMaORI_P*csrA*_*lb*_ were not different from those of their parental strains (Fig 3A and S6B Fig), suggesting that *csrA* was not essential for growth in *L*. *biflexa* in rich medium. However, the growth of Δ*csrA* was defective under starvation condition compared to its parental WT strain. There was a relatively slow lag phase before reaching a similar growth rate as that in WT in the stationary phase, indicating that *csrA* is required in the early phase of growth when nutrients are limited. However, the complemented strain was unable to fully restore the phenotype. This is probably due to the overexpression (3-fold increase) of *csrA* in *trans* in Δ*csrA* compared to the wild-type which may result in massive gene deregulation as shown by RNA-seq which had more than 500 differentially expressed genes (Fig 4B). Our data suggested that CsrA is required for growth of *L*. *biflexa* under starvation so that they can survive in the environment where nutrients are limited.

Several reports showed that there was an alteration of transcriptomic profile in *csrA* mutant strains and those strains support CsrA as a global gene regulator (Table 1). In *csrA* mutant of enterohemorrhagic *Escherichia coli* O157:H7, 641 genes were upregulated, and 703 transcripts (~15% of total genes) were downregulated compared to its parental WT strain [26]. A total of 239 genes (13.4% of total genes) showed different expression in *csrA* mutant of *B*. *burgdorferi* compared to its parental WT [23]. Surprisingly, only 3 genes (<1% of total genes) were differentially expressed in Δ*csrA* of *L*. *biflexa*, but only 1, LEPBIa1872 (*flaB3*), was significantly upregulated more than 1.5-fold change and its expression level could be restored in the complemented strain indicating that *csrA* is a repressor of *flaB3*. A small number of genes were detected by RNA-seq probably because of different mechanisms employed by CsrA to regulate its gene targets [63]. For example, CsrA post-transcriptionally regulates its gene targets by affecting their mRNA stability [35, 36, 77] or it can regulate its targets without any change in the number of target transcripts [76, 78]. As a result, such post-transcriptional control might not be detected by RNA-seq. Proteomic profiling may be necessary to investigate post-translational effects of CsrA as well as to identify its targets. In addition, we found that Δ*csrA* of *L*. *biflexa* grew slower than WT strain under starvation conditions. Thus, transcriptomic profiling of Δ*csrA* under starvation may yield more information on the target genes.

In contrast to Δ*csrA*, a higher number of genes were differentially expressed in the Δ*csrA*+pMaORI_P*csrA*_*lb*_ overexpressing CsrA in *L*. *biflexa* (Fig 4B). This finding might be a result of deregulation of other regulators as reported in some bacteria [63]. Presumably, CsrA exerts global regulation in *L*. *biflexa* when its expression reaches a substantial level. RNA-seq of WT demonstrated low *csrA* expression in rich medium (low total read/sample of *csrA* in S2 Table), therefore deletion of *csrA* might not result in major transcriptomic changes. In addition, other unknown factors might inhibit *csrA* expression at its native locus because the expression of *csrA* under its native promoter was significantly higher than WT (Fig 2B). Accordingly, we cannot exclude the possibility that CsrA is a global regulator in *L*. *biflexa* especially under the conditions that upregulate *csrA*. The impact of CsrA on expression of other genes under such conditions require further investigation.

Motility is one of the common traits regulated by CsrA. The alteration in motility affected by CsrA as well as the molecular mechanisms of CsrA that act on motility genes have been well documented in many bacteria [40, 79–84]. Flagellin genes have been reported as targets of CsrA in many bacteria. For example, CsrA bound to 5’untranslated regions of borrelial *flaB* at the consensus sequences overlapping the Shine Dalgarno sequence resulted in the translational block [51]. Hag protein, which shares ~47% amino acid identical to leptospiral *flaB3*, is regulated by CsrA using the same mechanism as *Borrelia* [78]. Our transcriptome analysis showed that *flaB3* is a potential target of CsrA. This was further confirmed by the presence of putative CsrA binding site in the promoter and gel shift assays which showed that there was a specific binding of rCsrA to the WT *flaB3* 5’untranslated region through the GGA conserved residues, which is consistent with a previous report [41]. In contrast, this finding is inconsistent with the results from the RNA-seq and EMSA results which showed that there were no differences in motility on soft agar, cell length, and velocity (Fig 3C–3E). While most bacteria harbor one flagellin component [85], *Leptospira* have 4 homologs of the flagellin FlaB in their genome [86]. The numbers of each FlaB in *L*. *interrogans* are approximately 14000, 2000, 300, and 3500 copies for FlaB1, FlaB2, FlaB3, and FlaB4, respectively [86]. Our RNA-seq results revealed that *flaB4* (LEPBIa1589) was the most transcribed *flaB*, more than 3-fold compared to other *flaB* transcripts (S2 Table). Apparently, FlaB3 (LEPBIa_1872), which is regulated by CsrA, is not a major FlaB protein, which could explain the absence of change in the motility phenotype. It is possible that *flaB2* is a target of *L*. *biflexa* CsrA because it was significantly upregulated in Δ*csrA* and its expression was restored in the complemented strain (Fig 5A), but the interaction was not investigated in this study. The putative CsrA binding site of *flaB2* shares high similarity to the *flaB3* binding site and harbors GGA motif (Fig 5B and S3 Table). However, although *flaB1* and *flaB4* genes contain a possible CsrA binding site (Fig 5B and S3 Table), no transcriptional change was observed. Other cooperating factors might be required for gene regulation. It is noteworthy to mention that the flagellar expression and motility phenotype observed here occurred in a nutrient-rich culture medium.

Because we were unable to generate a *csrA* mutant strain of *L*. *interrogans*, an overexpressing *csrA* strain was constructed. A 4-fold increase of *csrA* in *L*. *interrogans* resulted in poor motility on soft agar (Fig 7C), suggesting that *csrA* may regulate the motility of *L*. *interrogans*. Due to no alteration in cell length or velocity, other pathways regulated by CsrA may be responsible for this phenotype. These results indicated the crucial function of *csrA* in the pathogenic strain because motility is known to be a virulence factor of *Leptospira* [87–89].

The upregulation of *flaB* in *csrA* overexpressing strain of *L*. *interrogans* is in contrast to the downregulation in *csrA* overexpressing strain of *L*. *biflexa*, suggesting the distinct mechanisms of *csrA* among leptospiral strains. As previously reported, the mechanism of CsrA on motility regulation can be distinct in different bacteria. For example, the *csrA* mutants in *E*. *coli* and *S*. Typhimurium were non-motile and CsrA positively regulated the master operon in flagellum biosynthesis, *flhDC* [40, 90]. In contrast, RsmA, a CsrA homolog of *Erwinia carotovora*, negatively regulated *flhDC*, and the *rsmA* mutant was hypermotile [82]. In addition, CsrA in *Salmonella* regulated different motility genes compared to *E*. *coli* [38]. These results indicated that CsrA in different bacteria may have distinct effects on motility. For example, the same gene in different bacteria may have different mechanisms or control of different gene targets. Comparative transcriptomic and proteomic profiles of *csrA*-overexpressing *L*. *biflexa* and *L*. *interrogans* might give useful information on the global role of CsrA as well as different mechanisms of CsrA between these 2 species.

In conclusion, we characterized the role of CsrA in *Leptospira* spp. We found that *csrA* of the saprophyte *L*. *biflexa* is required for starvation response and repressed the expression of *flaB3* (*LEPBIa_1872*) without any change in motility phenotype. *L*. *biflexa* CsrA may exert a global effect under certain conditions that upregulate *csrA* expression. In contrast, overexpression of CsrA in pathogenic *L*. *interrogans* resulted in poor motility and CsrA may be an activator of *flaB1*, *flaB2*, and *flaB4* genes. This study suggested that pathways of gene regulation by CsrA may be different in bacteria belonging to the same genus, i.e., pathogenic and non-pathogenic *Leptospira* spp.

## Supporting information

S1 Fig Map of suicide vectors. Map of suicide vectors, *L*. *interrogans* serovar Manilae and *L*. *biflexa* serovar Patoc. These vectors have Km^R^ located between the flanking sequences of *csrA*.
(TIF)

S2 Fig Map of pMaORI used for complementation and overexpression. pMaORI containing *csrA* of *L*. *interrogans* serovar Manilae and *L*. *biflexa* serovar Patoc with its native promoter.
(TIF)

S3 Fig PCR confirmation of *csrA* mutant strain in *L*. *biflexa* serovar Patoc. (A) Genomic DNA of wild type and 16 selected transformants were prepared and amplified by PCR using specific primers that flanked sequences of *csrA* (Flk-L and Flk-R). (B) To confirm the absence of *csrA*, we amplified 2 transformants which were positive for double crossing over event using PCR with specific primers to the coding sequence of *csrA* (ORF-L and ORF-R).
(TIF)

S4 Fig GO term enrichment analyses on DEGs identified from WT and Δ*csrA+*pMaORI_P*csrA*_*lb*_. The significant enriched biological process for downregulated genes in the complemented strain are shown. No enriched GO terms were found in the upregulated genes in the complemented strain.
(TIF)

S5 Fig Recombinant CsrA production. PCR products of complete sequences of *csrA* either from *L*. *interrogans* or *L*. *biflexa* were cloned into pRSET-C expression vector, transformed in *E*. *coli* BL21 (DE3) pLysS, and induced the expression IPTG. Purified N-terminal 6x His tag recombinant CsrA was subjected to 15% SDS-PAGE and stained with Coomassie Brilliant Blue R-250. Separated recombinant proteins were blotted onto a nitrocellulose membrane, detected with mouse monoclonal antibody against 6×His tag (primary antibody) and HRP-conjugated anti-mouse IgG (secondary antibody) using Amersham ECL Western Blotting Detection Reagent.
(TIF)

S6 Fig Overexpression of *csrA* in *L*. *biflexa*. (A) Overexpression of *csrA* in *L*. *biflexa*. To confirm overexpression of *csrA*, RNAs were extracted from each *Leptospira* strain and subjected to RT-qPCR. Results obtained from 3 independent cultures were presented as relative fold changes ± SEM. *cysK* was used for normalization. (***) indicated p-value <0.001. The growth curve of *L*. *biflexa*. The 2×10^6^ cells of each bacterial strain were grown in 10 mL of regular EMJH or 5-fold diluted EMJH in water, (B) represented growth in regular EMJH and (C) growth in 5-fold diluted EMJH. OD_420_ measurement for growth was performed every 24 h. Results obtained from 3 independent experiments are expressed as Mean ± SEM. (D) Soft agar assay of *L*. *biflexa*. *Leptospira* OD_420_ = 0.1 were inoculated onto 0.6% semisolid EMJH plate and incubated at 30°C. (E) Measurement of cell length of *L*. *biflexa* (F) Measurement of velocity of *L*. *biflexa*. Late exponential phase of *Leptospira* grown in EMJH medium were measured for cell length and velocity under a dark-field microscope using cellSens software (OLYMPUS).
(TIF)

S1 File (PDF)

S1 Table Primers used in this study. (XLSX)

S2 Table Significantly deregulated genes in the Δ*csrA* and Δ*csrA*+pMaORI_P*csrA*_*lb*_ with log2FC > ± 0.5 cut-off and adjusted p-value of <0.05. (XLSX)

S3 Table Analysis of *flaB* 5’ untranslated region. ^a^ Gene, ORF ID, Product, and Distance to the start codon are according to *Leptospira biflexa* serovar Patoc Patoc 1 and *L*. *interrogans* serovar Manilae strains. UP-MMC-NIID LP was obtained from MicroScope Microbial Genome Annotation & Analysis Platform; https://mage.genoscope.cns.fr/microscope/home/index.php. Underlined letters represented mismatch nucleotide compared to the consensus sequence. Bold letters represented the Shine-Dalgarno sequence.
(XLSX)
