# Supplementary figures and images for: Investigating the role of the carbon storage regulator A (CsrA) in Leptospira spp
Source: PLoS One. 2021 Dec 13;16(12):e0260981. doi: 10.1371/journal.pone.0260981 (PMC8668096; doi:10.1371/journal.pone.0260981)

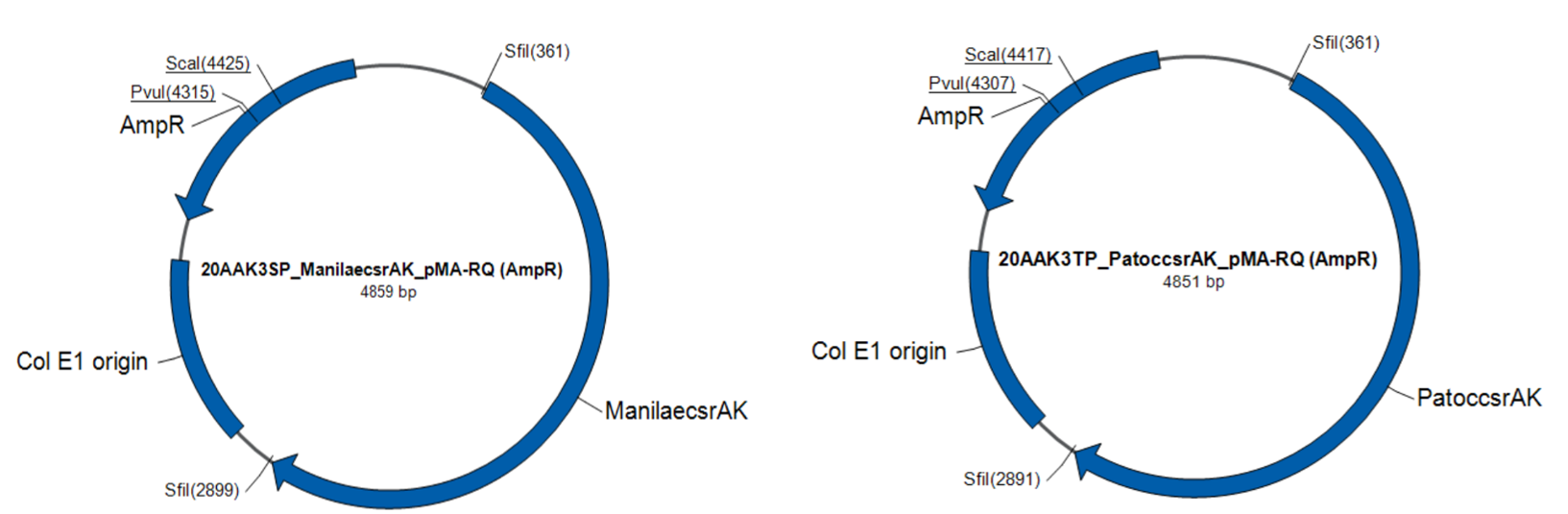

Supplement: S1 Fig — Map of suicide vectors, L. interrogans serovar Manilae and L. biflexa serovar Patoc. These vectors have KmR located between the flanking sequences of csrA. (TIF) [file pone.0260981.s001.tif]

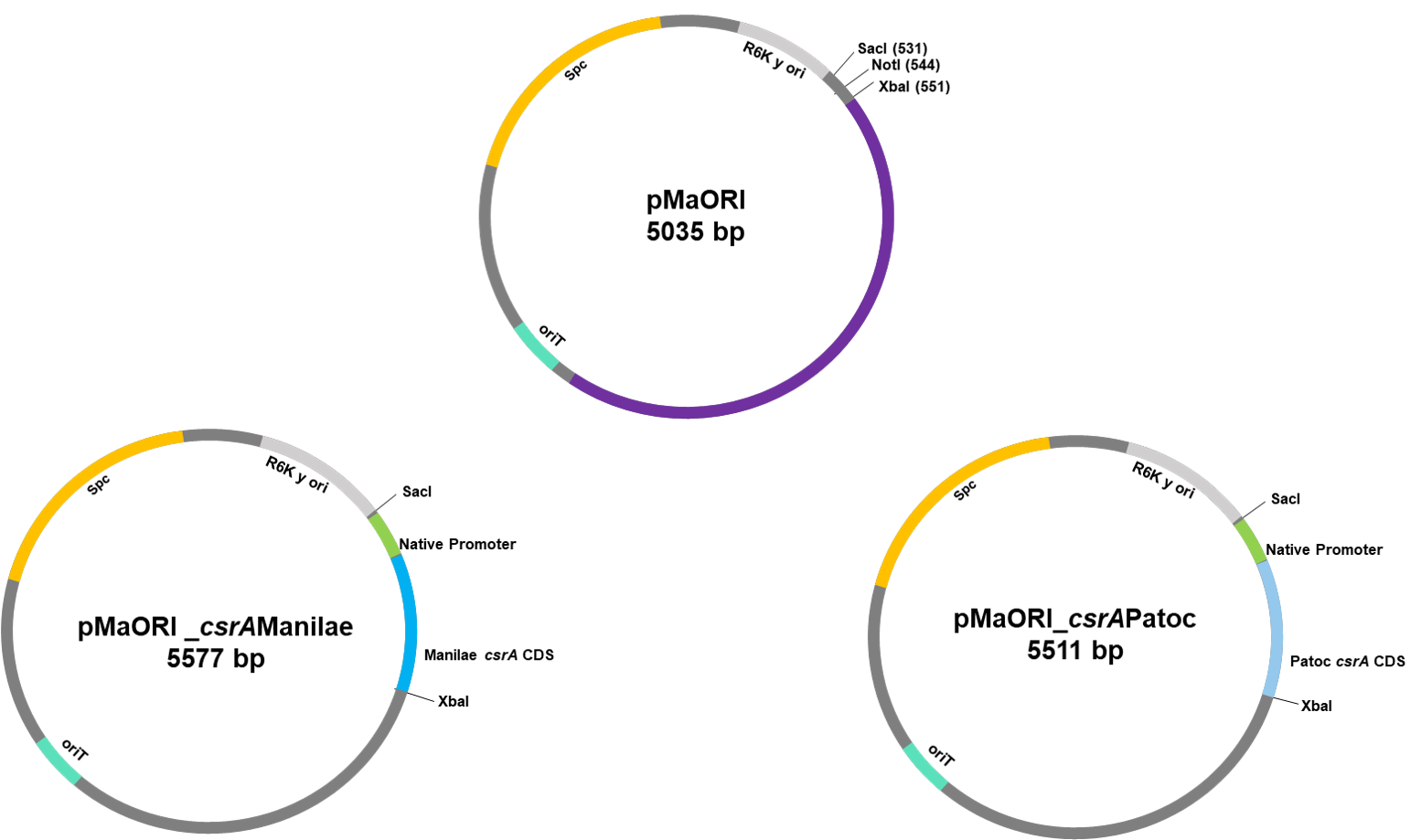

Supplement: S2 Fig — pMaORI containing csrA of L. interrogans serovar Manilae and L. biflexa serovar Patoc with its native promoter. (TIF) [file pone.0260981.s002.tif]

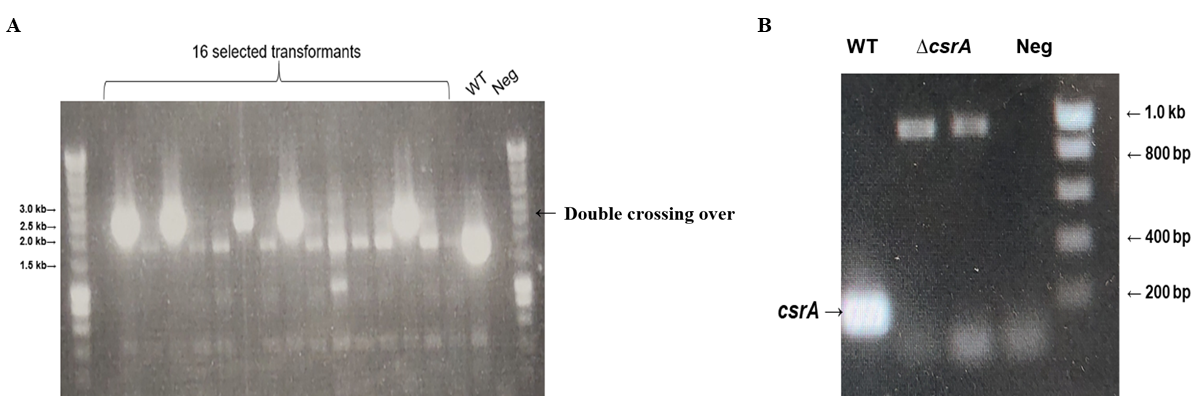

Supplement: S3 Fig — (A) Genomic DNA of wild type and 16 selected transformants were prepared and amplified by PCR using specific primers that flanked sequences of csrA (Flk-L and Flk-R). (B) To confirm the absence of csrA, we amplified 2 transformants which were positive for double crossing over event using PCR with specific primers to the coding sequence of csrA (ORF-L and ORF-R). (TIF) [file pone.0260981.s003.tif]

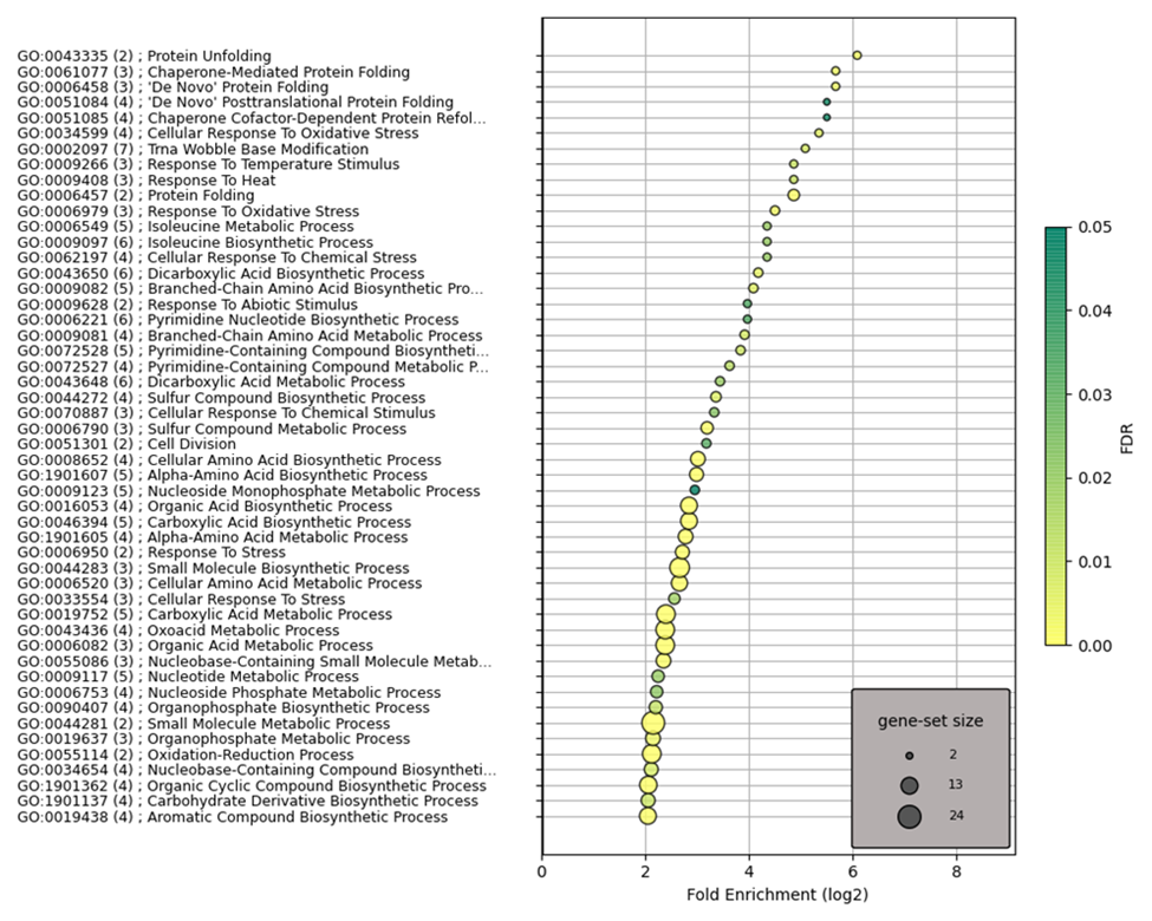

Supplement: S4 Fig — The significant enriched biological process for downregulated genes in the complemented strain are shown. No enriched GO terms were found in the upregulated genes in the complemented strain. (TIF) [file pone.0260981.s004.tif]

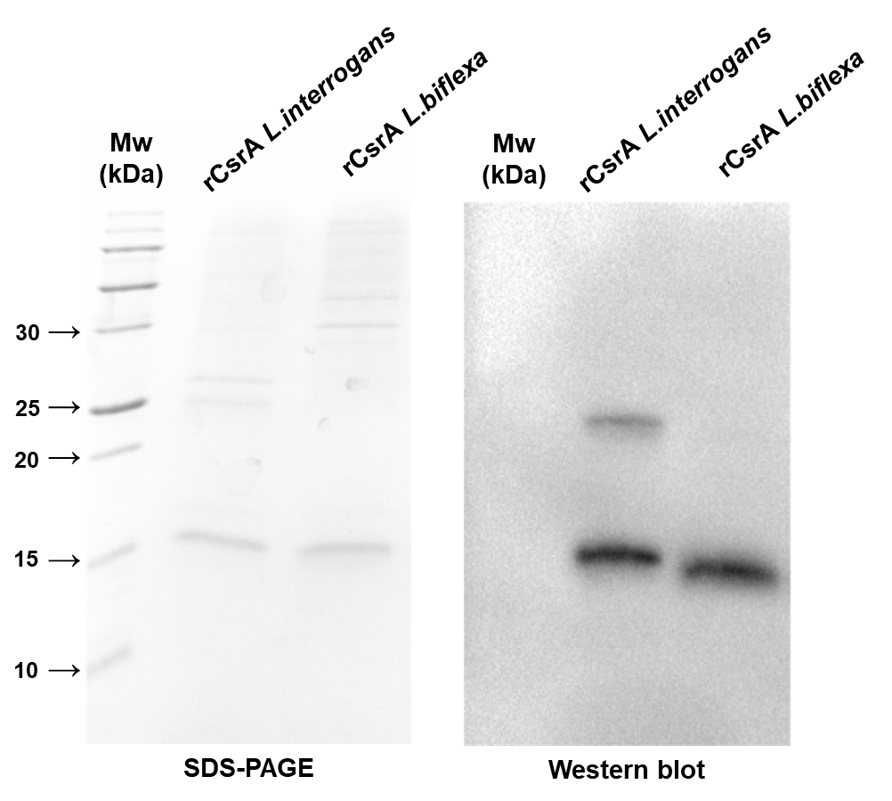

Supplement: S5 Fig — PCR products of complete sequences of csrA either from L. interrogans or L. biflexa were cloned into pRSET-C expression vector, transformed in E. coli BL21 (DE3) pLysS, and induced the expression IPTG. Purified N-terminal 6x His tag recombinant CsrA was subjected to 15% SDS-PAGE and stained with Coomassie Brilliant Blue R-250. Separated recombinant proteins were blotted onto a nitrocellulose membrane, detected with mouse monoclonal antibody against 6×His tag (primary antibody) and HRP-conjugated anti-mouse IgG (secondary antibody) using Amersham ECL Western Blotting Detection Reagent. (TIF) [file pone.0260981.s005.tif]

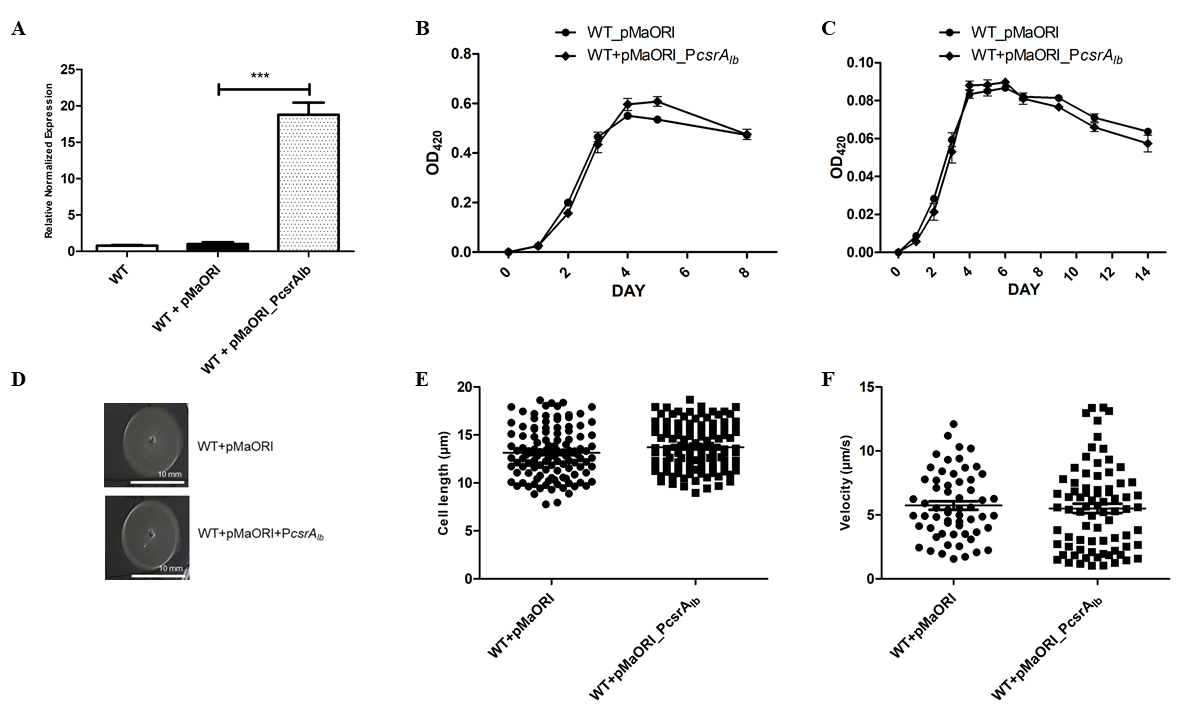

Supplement: S6 Fig — (A) Overexpression of csrA in L. biflexa. To confirm overexpression of csrA, RNAs were extracted from each Leptospira strain and subjected to RT-qPCR. Results obtained from 3 independent cultures were presented as relative fold changes ± SEM. cysK was used for normalization. (***) indicated p-value <0.001. The growth curve of L. biflexa. The 2×10^6^ cells of each bacterial strain were grown in 10 mL of regular EMJH or 5-fold diluted EMJH in water, (B) represented growth in regular EMJH and (C) growth in 5-fold diluted EMJH. OD420 measurement for growth was performed every 24 h. Results obtained from 3 independent experiments are expressed as Mean ± SEM. (D) Soft agar assay of L. biflexa. Leptospira OD420 = 0.1 were inoculated onto 0.6% semisolid EMJH plate and incubated at 30°C. (E) Measurement of cell length of L. biflexa (F) Measurement of velocity of L. biflexa. Late exponential phase of Leptospira grown in EMJH medium were measured for cell length and velocity under a dark-field microscope using cellSens software (OLYMPUS). (TIF) [file pone.0260981.s006.tif]
