# Supplementary material for: Investigating the role of the carbon storage regulator A (CsrA) in Leptospira spp
Source: PLoS One. 2021 Dec 13;16(12):e0260981. doi: 10.1371/journal.pone.0260981 (PMC8668096; doi:10.1371/journal.pone.0260981)
Supplement: S1 File — (PDF) [file pone.0260981.s007.pdf]

Fig 6B original image

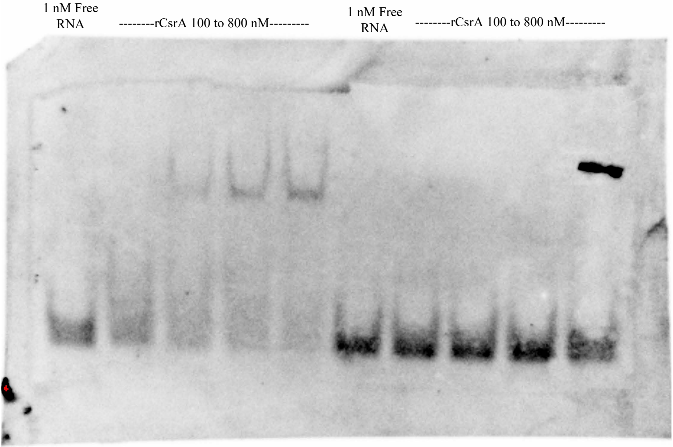

Fig 9C original image

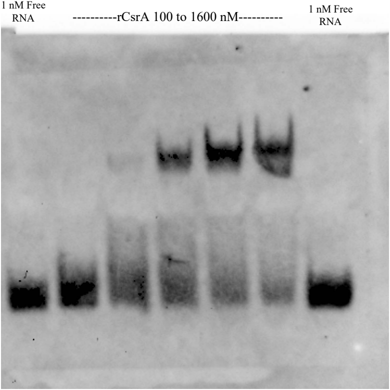

Fig 9D original image

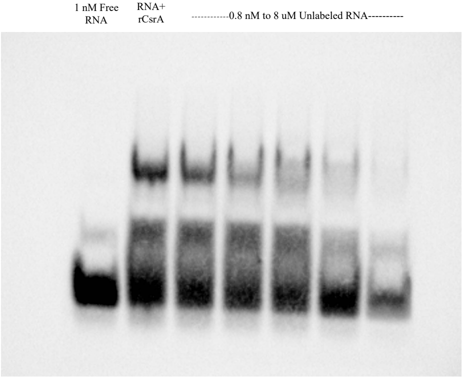

S5 Fig original image

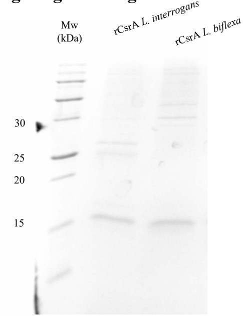

SDS-PAGE

S5 Fig original image

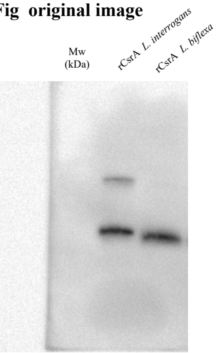

Western blot
